# Applications of Network Pharmacology in Traditional Chinese Medicine Research

**DOI:** 10.1155/2020/1646905

**Published:** 2020-02-14

**Authors:** Zhuchen Zhou, Bing Chen, Simiao Chen, Minqiu Lin, Ying Chen, Shan Jin, Weiyan Chen, Yuyan Zhang

**Affiliations:** ^1^School of Life Science, Zhejiang Chinese Medical University, Hangzhou, Zhejiang 310053, China; ^2^School of Basic Medicine, Zhejiang Chinese Medical University, Hangzhou, Zhejiang 310053, China

## Abstract

Human diseases, especially infectious ones, have been evolving constantly. However, their treatment strategies are not developing quickly. Some diseases are caused by a variety of factors with very complex pathologies, and the use of a single drug cannot solve these problems. Traditional Chinese Medicine (TCM) medication is a unique treatment method in China. TCM formulae contain multiple herbs with multitarget, multichannel, and multilink characteristics. In recent years, with the flourishing development of network pharmacology, a new method for searching therapeutic drugs has emerged. The multitarget action in network pharmacology is consistent with the complex mechanisms of disease and drug action. Using network pharmacology to understand TCM is an emerging trend.

## 1. Network Pharmacology

With the development of the information age, various network technologies are being continuously developed. Based on system biology, bioinformatics, and high-throughput histology, network pharmacology, which integrates pharmacology and information network, is gaining momentum [1–3]. The concept of network pharmacology was first proposed by Andrew L. Hopkins in 2007 [4]. It combines network biology with polypharmacology, based on the poor efficacy of highly selective single-target drugs [5]. Through network pharmacology, we can directly identify drugs and disease targets from a large amount of data and understand the mechanisms and pathways between them [6–9]. It is an effective method. Nowadays, the application spectrum of network pharmacology is expanding and includes exploring the basic pharmacological effects of drugs on diseases and their mechanisms [10, 11], analyzing the theory of TCM [12, 13], and studying the application of TCM [14].

Common tools of network pharmacology include databases like DrugBank [15], STITCH [16], and TCM chemical information databases [17] containing data on drug molecules, databases related to active ingredients such as PubChem [18] and ChEMBL [19], KEGG [20], and Target database, gene-related databases like OMIM [21], protein-related databases like HPRD [22], BioGRID [23], and DIP [24], and biomolecular interaction databases like HPRD, BIND [24], DIP, HAPPI [25], MINT [23], STRING [26], and PDZBase [27]. They can all be used to find the necessary information. Besides these databases, appropriate tools are needed [28–30], such as Cytoscape, Pajek, VisANT, GUESS, WIDAS, PATIKA, PATIKAweb, and CADLIVE. At present, in the field of TCM research, Cytoscape, GUESS, Pajek, and VisANT are the most widely used network analysis software.

## 2. Application in Traditional Chinese Medicine

Chinese medicine is a treasure of the Chinese nation and it has a long history. After thousands of years of tempering and testing, it has been improved and perfected and eventually formed into a unique and complete theoretical system of Chinese medicine which is different from western medicine. The use of TCM formulae is the main means of TCM treatment, and TCM formulae prescription is multicomponent, containing many medicinal flavors, and has the advantages of being a multitarget, multichannel, and multilink system. However, due to the complex composition, the TCM material basis and mechanism is unclear just like its toxicity and side effects and we are unable to determine the specific active ingredients. Certain problems such as the interaction between targets bring a level of uncertainty into the TCM system and make it unacceptable to the natives and nonnatives. Therefore, it must be combined with network pharmacology to make TCM more effective [31–34]. With the development of human society and the change of surroundings, human diseases have gradually transformed from infectious diseases to chronic diseases including cardiovascular diseases, diabetes mellitus, and tumors. These diseases are often not only caused by a single factor but also caused by several factors simultaneously, which coincides with the use of TCM prescriptions [1, 2]. The major features of TCM include a holistic view, treatment based on TCM syndrome differentiation, and prescription medication, which are in line with the overall systematic characteristics of network pharmacology [35, 36].  Table 1 mainly lists the application of network pharmacology in Chinese medicine treatment of diseases.

### 2.1. Traditional Chinese Medicine Syndrome Research

The research on ZHENG for many years shows that it is not enough to express ZHENG by a single physiological and biochemical index as it is a complex system composed of many factors [1]. The biological basis of TCM syndrome theory and the relationship between disease syndromes are unclear, and this lack of clarity has become a stumbling block in the application of TCM prescriptions to modern medicine [3]. Therefore, it is necessary to adopt a new way to understand the TCM syndromes in complex systems. The application model of “disease-phenotype-gene-drug” in network pharmacology can guide the study of TCM syndromes [45].

Niu et al. [37] screened four common medicines for treating rheumatoid arthritis (RA) of heat syndrome using network pharmacology and PubChem. The four common medicines are *Rhizoma Anemarrhenae*, *Achyranthes*, *Cortex phellodendri*, and Atractylodes Atractylodis. TCM target proteins of RA heat syndrome were identified using PubChem. Four identical biological pathways were found in the constructed molecular network of heat syndrome rheumatoid arthritis (RA), and the molecular interaction network of target proteins of TCM syndromes includes GM-CSF signaling, CTLA4 signaling in cytotoxic T lymphocytes, T-cell receptor signaling, and CD28 signaling in T helper cells. These four pathways are closely related to cellular immune response and inflammatory response indicating that these pathways are important molecular biological mechanisms for treating RA heat syndrome with TCM.

Chen et al. [38]selected potential targets of TCM for the treatment of cough variant asthma (CVA) through network pharmacology, constructed a network of medicinal materials and ingredients, and explained their efficacy by searching for relevant channels. The experimental verification system showed that “FengXie FanFei,” “FeiQiShiXuan,” and “QiDao LuanJi” have certain effects on the regulation of different biological processes. The therapeutic effects of the three factors are achieved by the metabolic processes of steroids and the pathways of EGFR, VEGF, Gn-RH, and so on. Besides, they also play a regulatory role in ion conduction and transport.

Li et al. [46] studied ZHENG with the help of neuroendocrine-immune networks (NEI) through computational analysis and animal experiments. It showed that cold ZHENG and hot ZHENG are two different states. Network pharmacology and other methods were used to analyze and compare the results. It was found that hormones play a key role in cold ZHENG and immune factors play a key role in hot ZHENG. The two may be connected by neurotransmitters. It also provides a theoretical basis for the design of a novel therapeutic method based on the molecular basis of ZHENG in the future.

Chen et al. [47] studied and analyzed the excessive, excessive-deficient, and deficient syndromes of hepatocellular carcinoma (HCC) by studying the data of miRNA array. It was found that the deficient syndrome (LKYDS) was first transformed into excessive-deficient syndrome (LDSDS) and then into a parallel branch of excessive syndrome (LGDHS). It was indirectly inferred that the excessive-deficient syndrome is more closely related to cancer than deficient syndrome and that miRNAs can be treated as potential molecular markers as they play an important role in it.

### 2.2. Compatibility of Traditional Chinese Medicine

TCM was initially used in the form of a single drug and it gradually evolved into a multidrug compatibility treatment. With the rapid development of modern science and technology, many diseases have been confirmed to be caused by a combination of factors. Therefore, the “multitarget multicomponent multidisease” treatment of diseases has become a trend and it involves using multiple drugs to treat diseases. Based on network pharmacology, data screening can prove the effectiveness of Chinese medicine in treating diseases.

Liu et al. [48] screened 133 targets from *Cistanche tubulosa* through drug targeting process combined with drug similarity evaluation using network pharmacology. The top ten drug combinations were listed by the algorithm. Probability Ensemble Approach (PEA) was selected to construct a composite target pathway.

Wu et al. [39] built an Acute Myocardial Ischemia (AMI) specific Organism Disturbed Network (AMI-ODN) by collecting disease-related genes, proteins, and other data with the help of network pharmacology and developed a Network Recovery Index for Organism Disturbed Network (NRI-ODN), which indirectly demonstrated the therapeutic effect of QiShenYiQi (QSYQ) by detecting the ability of AMI network to restore normal level under the disturbed state. The experimental results showed that the overall NRI-ODN score of QSYQ was higher than that of all the other single drugs. This indicates that QSYQ has a certain effect on the treatment of acute myocardial ischemia. It also shows that the cumulative effect of TCM formulae is better than that of a single Chinese medicine. This experiment also found that *Salvia miltiorrhiza* and *Astragalus membranaceus* play an important role in apoptosis and inflammation through network pharmacology in the pathway level. All of these provide a theoretical basis for subsequent experiments.

Sheng et al. [49] found that 41 protein targets related to fibrinolysis, coagulation factors, and platelet aggregation are related to 22 components of compound Fufang Xueshuantong (FXST) by means of network pharmacology. It is predicted that therapeutic conditions can be achieved through the interaction between the targets and components. FXST capsules contain multiple chemical components that can act on the same disease target and interact to treat thrombosis. An experimental validation was conducted in a rat model of disseminated intravascular coagulation (DIC) induced by lipopolysaccharide, and the results showed that FXST has a therapeutic effect on DIC.

Shen et al. [50] determined the contents of aconitine, hypaconitine, and neoaconitine in rat plasma after oral administration of aconitine decoction by high-performance liquid chromatography-mass spectrometry (HPLC-MS) and analyzed their pharmacokinetic changes. It was found that the Cmax (peak concentration) and AUC (area under drug time curve) of the three components decreased in varying degrees, while MRT (mean dwell time) and *t*1/2 (end elimination half-life) were prolonged in varying degrees after combining licorice and aconite decoction. It shows that the combination of licorice and aconite slows down the absorption of toxic substances and has a certain attenuation effect, and the combined decoction has better attenuation effect.

Yang et al. [40] established a drug database on the basis of network pharmacology. They also searched and identified the top five medicines in the China Biology Medicine disc (CBMdisc) for the treatment of osteoarthritis. These medicines are *Achyranthes bidentata*, *Angelica sinensis*, licorice, *Eucommia ulmoides*, and *Paeonia lactiflora* and verified their core compatibility. The relevant genes of osteoarthritis and target proteins of the above drugs were then discerned. The method of ingenuity pathway analysis (IPA) was used to construct the molecular network. Finally, it was concluded that the compatible drugs play an important role in the treatment of osteoarthritis through the influence of cellular immune pathway and related cell proliferation, growth, and apoptosis pathway.

### 2.3. Drug Discovery

As time goes on, diseases are constantly changing, and requirement for the research and development of new drugs is constantly increasing [51]. New drugs are required not only for treatment but also for prevention [52]. Since it takes too long for a new drug to be developed and released to the FDA, we can adopt the new method of using old drugs or using a single molecule to correspond to multiple targets. Through the method of network pharmacology and the use of a large database of drug targets corresponding to the relevant diseases, it was found that a drug can play a role in the treatment of a variety of diseases. This corroborates the hypothesis that we can treat many diseases with one medicine [53].

Tang et al. [54] found that three substances in *Hypericum patulum*, *Sedum acre*, and *Tripterygium wilfordii* can bind to zinc binding sites of Matrix Metalloproteinase-9 (MMP-9), which plays an important role in acute skin inflammation. This method can be used to avoid the side effects of tetracycline and the curative effect can be improved by using high-throughput virtual screening method based on network pharmacology. This may be developed as a new treatment for acute skin inflammation later.

Using network pharmacology, Hu and Sun [41] searched 28 Chinese herbal medicines for the treatment of type 2 diabetes mellitus (T2DM) in Medicinal Plants Database (MPDB) and TarNet, and 5493 targets were identified. In the constructed interaction network of type 2 diabetes mellitus (T2MD) disease protein and protein-protein interaction network (PPI network), reasonable intervention was carried out with special algorithms. Network intervention score (NIS) and *P* scores were used to evaluate the effective TCM combination as a new formula for T2MD treatment.

Klee et al. [55] summarized the method of using a single drug to treat epilepsy using network pharmacology. Based on the analysis of a large amount of data, drug combination tolerance experiments were carried out which laid the foundation for the development of new drugs for the treatment of epilepsy.

Tang et al. [56] identified 37 potential targets related to AR in Mahuang Fuzi Xixin Decoction (MFXD) by means of network pharmacology, oral bioavailability prediction, multiple drug target prediction, and network analysis and predicted that four of them were closely related to anti-inflammatory effects. It provides a theoretical basis for the treatment of new drugs for AR.

Gu et al. [57] through the study of TCM for cardiovascular disease (CVD) and its mechanism of action constructed a systematic database for drug discovery based on natural products. This website can carry out virtual screening and drug discovery of Chinese herbal medicine related to CVD treatment and can find the relevant targets and clinical markers of Chinese herbal medicine and CVD diseases. Hence, a platform for the research and development of new drugs for the treatment of CVD was provided.

Fang et al. [42] combined network pharmacology-based method with large-scale text mining, target prediction, drug-likeness filtering, and network analysis, mass screening was conducted in PubMed, and 10 herbs with the significant correlations with Alzheimer's disease (AD) were selected. After the drug-likeness filtering and clustering analysis, compound-target (C-T) and target-pathway (T-P) networks were built to explain the mechanism for anti-AD herbs. Compared with previous studies, not all 10 drugs were originally used in the treatment for AD, which provides ideas for new treatments for AD.

### 2.4. Chinese Medicine Formula

The compound is a combination prescription based on the overall view, syndrome differentiation, and treatment according to the principle of Junchen Zuoshi, Qiqinghehe, and so on [1, 58]. Many TCM formulae have been proven to have a therapeutic effect on diseases. The Chinese medicine in the TCM formula is selected and confirmed by network pharmacology.

Zeng et al. [59] collected the compounds of *Astragalus* Salvia compound (ASC) and identified their targets through network pharmacology and found pregnancy-induced hypertension syndrome (PIH) targets in Genecards and OMIM at the same time. Based on this, four new networks were constructed to conclude that ASC could directly regulate genes and biological processes related to “endothelial cell activation and injury” and “placental or trophoblastic cell ischemia,” and it was concluded that ASC could indirectly treat PIH.

Jiang et al. [60] studied the anti-inflammatory mechanism of Danhong injection (DHI) through network pharmacology and high-throughput screening (HTS). The anti-inflammatory components of DHI were screened by cell experiments. They found the potential active ingredients including danshensu, caffeic acid, protocatechuic acid, protocatechuic aldehyde, safflor yellow A, hydroxysafflor yellow A, salvianolic acid A, salvianolic acid B, and salvianolic acid C. It was found that DHI can inhibit inflammation by inhibiting NF-*κ*B. The inhibitory effect of the active ingredient SAC in DHI on NF-*κ*B was reported for the first time.

Tao et al. [61] constructed a computational biology method based on the combination of network pharmacology and pharmacochemical structural genomics and other new systems. It was found that 32 potential targets of Chinese herbal Radix Curcumae formula were related to cardiovascular and cerebrovascular diseases (CCVD), among which *Radix Curcumae* and Fructus Gardeniae had the most common targets. It has been proven by experiments that a combination of TCM and animal formula can improve the efficacy. In addition, by studying the target of Radix Curcumae formula under network verification, it was found that it can also play a therapeutic role in tumor and metabolic diseases.

Zeng et al. [43] found potential targets of HER2-positive breast cancer through network pharmacology and obtained 13 pathways related to HER2-positive breast cancer. This study confirmed the mechanism of Yanghe Decoction (YHD) in the treatment of HER2-positive breast cancer and indirectly proved the effectiveness of YHD Chinese herbal combination. Ras signaling pathway, PI3K-Akt signaling pathway, MAPK signaling pathway, HIF-1 signaling pathway, VEGF signaling pathway, and other signaling pathways were also found to be connected. This provides a new theoretical basis for the future study of YHD as a method of treatment for HER2-positive breast cancer.

Pang et al. [44] explored the mechanism of Naodesheng (NDS) formula for the treatment of Alzheimer's disease (AD) by constructing constituent-target network, constituent-target-target network, and target-biological pathway network with the help of network pharmacology. Oral bioavailability, blood-brain barrier permeability, and drug likeness were evaluated to predict the results through Discovery Studio 4.1. Based on the prediction of multitarget compounds from NDS formula and the critical protein targets in the AD formation mechanism, Pang et al. built a network system. The results suggested that there was indeed a potential correlation between NDS formula and the treatment of AD, which provided a basis for future studies.

## 3. Conclusion

The major features of Traditional Chinese Medicine (TCM) include a holistic view, treatment based on TCM syndrome differentiation, and prescription medication, which are in line with the overall systematic characteristics of network pharmacology. The characteristics of TCM research fit well with the characteristics of network pharmacology. Compared with other research methods, this characteristic is the advantage of this method. Taking Chinese medicine as the research object, the method of network pharmacology was used to study its components, then predict the target, analyze the pathway, and finally perform experimental verification for the disease to explore the mechanism of action of Traditional Chinese Medicine treatment.

Regarding the above articles, many scholars have provided new ideas and methods for the treatment of some diseases through the application of network pharmacology. The research on the mechanism of Traditional Chinese Medicine to treat diseases has made the use of Traditional Chinese Medicine more precise. In the face of disease, there are more previously unused drugs that can be used or drugs that are already used to treat another disease. However, due to the many aspects studied by scholars and the conditions which are different, the reliability of the results needs to be improved [51].

In recent years, network pharmacology has received extensive attention as an emerging discipline. However, it is still in the early stages of development and has many problems. Firstly, appropriate computing software has not been developed. The systematic screening, integration, and processing of data is another problem. The data on various drugs, genes, proteins, and so on are not comprehensive. The databases on Chinese herbal medicines, proprietary Chinese medicines, and TCM are incomplete, and their accuracy and integrity cannot be guaranteed [51, 62]. Network pharmacology uses computer network screening to achieve target selection, and many results do not have enough experimental data support [63]. The known compound targets do not fully correspond to the complex mechanism of TCM formulae [64]. However, the number of disease targets and drug target proteins are gradually increasing, and the understanding of these mechanisms is becoming more and more profound. Computer network software and calculation methods are being constantly updated; more corresponding clinical experiments and network pharmacology research results correspond to each other, which greatly increases the reliability of the results. These may lead to more widespread application of network pharmacology.

## Figures and Tables

**Table 1:** Application of network pharmacology in Chinese medicine.

Applications	Databases	Authors	Disease
Traditional Chinese Medicine syndrome research	CBM, PubChem, MetaDrug, and David	Niu et al. [37]	Rheumatoid arthritis (RA)
		Chen et al. [38]	Cough variant asthma (CVA)
Compatibility of Traditional Chinese Medicine	KEGG, HPRD, BioGRID, and PubChem	Wu et al. [39]	Acute myocardial ischemia (AMI)
		Yang et al. [40]	Osteoarthritis (OA)
Drug discovery	BioGRID, BIND, DIP, HPRD, MINT, MPDB, TarNet, KEGG, HIT, TCMSP, and TCMID	Hu and Sun [41]	Type 2 diabetes mellitus (T2DM)
		Fang et al. [42]	Alzheimer's disease (AD)
Chinese medicine formula	TCMSP, PharmMapper, UniProtKB, OMIM, STRING, DrugBank TCM Database, Chinese Natural Product Database, TCMSP, and TTD	Zeng and Yang [43]	HER2-positive breast cancer
		Pang et al. [44]	Alzheimer's disease (AD)
